# Disturbing dreams and dementia incidence across diverse cohort studies: A COSMIC collaboration study

**DOI:** 10.1111/pcn.70046

**Published:** 2026-03-17

**Authors:** Darren M. Lipnicki, Ashleigh S. Vella, Erico Castro‐Costa, Sergio Luis Blay, Maria Fernanda Lima‐Costa, Karen Ritchie, Elena Rolandi, Annalisa Davin, Michele Rossi, Ki Woong Kim, Ji Won Han, Dae Jong Oh, Ding Ding, Qianhua Zhao, Xiaowen Zhou, Yen‐Ching Chen, Jen‐Hau Chen, Perminder S. Sachdev, for Cohort Studies of Memory in an International Consortium (COSMIC)

**Affiliations:** ^1^ Centre for Healthy Brain Ageing (CHeBA), Discipline of Psychiatry and Mental Health, School of Clinical Medicine, Faculty of Medicine and Health UNSW Sydney Sydney New South Wales Australia; ^2^ Center for Studies in Public Health and Aging (NESPE) Belo Horizonte Brazil; ^3^ Department of Psychiatry Federal University of São Paulo São Paulo Brazil; ^4^ René Rachou Institute, Fiocruz Minas Belo Horizonte Brazil; ^5^ Institut for Neurosciences of Montpellier (INM) National Institute for Health and Medical Research (INSERM) Montpellier France; ^6^ Golgi Cenci Foundation Abbiategrasso Italy; ^7^ Department of Neuropsychiatry Seoul National University Bundang Hospital Seongnam Republic of Korea; ^8^ Department of Psychiatry Seoul National University College of Medicine Seoul Republic of Korea; ^9^ Department of Brain and Cognitive Science Seoul National University College of Natural Sciences Seoul Republic of Korea; ^10^ Institute of Human Behavioral Medicine Seoul National University Medical Research Center Seoul Republic of Korea; ^11^ Workplace Mental Health Institute, Kangbuk Samsung Hospital Sungkyunkwan University School of Medicine Seoul Republic of Korea; ^12^ Institute of Neurology, Huashan Hospital Fudan University Shanghai China; ^13^ Institute of Epidemiology and Preventive Medicine, College of Public Health National Taiwan University Taipei Taiwan; ^14^ Department of Geriatrics and Gerontology National Taiwan University Hospital Yunlin Branch Yunlin Taiwan

**Keywords:** Alzheimer's disease, bad dreams, dementia, incidence, nightmares

## Abstract

**Aim:**

Distressing dreams were previously reported to predict future all‐cause dementia among predominantly white US participants aged 79–89 years, particularly in men. We investigated whether disturbing dreams (nightmares and bad dreams) were associated with all‐cause and Alzheimer dementia (AD) among individuals aged 60–89 years from diverse international regions.

**Methods:**

Data were from six longitudinal cohort studies across Brazil, China, France, Italy, South Korea, and Taiwan (*n* = 10,238, 42.5% men). Cox regressions with a random effect for study investigated associations between disturbing dreams and incident dementia, with all participants and stratified separately by sex and baseline age. Analyses examined (i) any disturbing dreams and (ii) disturbing dreams at least once a week. Fully adjusted analyses included three studies with covariates for sleep problems, medications, mental and physical health, cognition, and APOE ε4 status.

**Results:**

Disturbing dreams were reported by 24.2% overall and all‐cause dementia, and AD incidence was 10.8 and 5.3 per 1000 person‐years, respectively. In fully adjusted analyses, having any disturbing dreams was associated with increased incidence of all‐cause dementia among 60–69‐year‐olds (hazard ratio [HR] 3.93, 95% confidence interval [CI] 1.32–11.67). There were no significant effects for older individuals. In fully adjusted sex‐stratified analyses, having disturbing dreams at least once a week was associated with AD only among men (HR 3.59, 95% CI 1.44–8.96).

**Conclusions:**

We found some evidence for disturbing dreams being associated with incident all‐cause dementia among individuals aged 60–69 years and with AD among men. The mechanisms potentially underlying these associations remain to be clarified.

The International Classification of Sleep Disorders describes nightmares as ‘extended, extremely dysphoric, and well‐remembered dreams that usually involve threats to survival, security, or physical integrity’ (p. 315)^1^. Nightmares are a type of disturbing dream distinguished from other types by waking the sleeper, but not by their degree of emotional intensity.^2^ Indeed, research questions employing any relevant term (e.g. nightmares, bad dreams) are thought to capture disturbing dreams that waken the sleeper as well disturbing dreams that do not waken the sleeper^2^, ^3^, ^4^ and we use ‘disturbing dreams’ as an umbrella term for these phenomena.

Disturbing dreams are risk factors for poor health, including mental health conditions like depression^5^ and physical health conditions like cardiovascular disease.^6^ Disturbing dreams have also been found to predict future neurodegenerative conditions, including Parkinson's disease (PD)^4^ and dementia with Lewy bodies (DLB).^7^ Evidence suggests that disturbing dreams are also associated with an increased risk of cognitive decline and dementia more broadly, including disturbing dreams in mid‐life being associated with poorer late‐life cognitive scores.^8^ Another study found that middle‐aged and older adults reporting at least one disturbing dream per week had greater chances of cognitive decline and incident dementia, respectively, with these effects significant only among men.^9^

Previous explanations linking disturbing dreams to neurodegenerative risk have drawn upon a neurocognitive model of nightmares that proposes fear memories are extinguished via activation and recontextualization during dreaming.^10^ This purportedly involves regulation of amygdala activity by the medial prefrontal and anterior cingulate cortices, and it has been suggested that neurodegeneration of frontal regions may prevent the downregulation of negative emotions in dreams.^4^, ^9^, ^11^ However, there are at least three additional potential mechanisms linking disturbing dreams to future dementia.

The associations between disturbing dreams and future PD and DLB^4^, ^7^ are complemented by around 80% of PD dementia (PDD) and DLB patients reporting disturbing dreams.^12^ PD, PDD, and DLB are characterized by Lewy bodies, intracellular aggregates of the amino acid alpha‐synuclein.^13^ Many Alzheimer dementia (AD) cases have Lewy bodies concomitant with beta‐amyloid plaques and phosphorylated tau protein tangles,^14^ suggesting that Lewy bodies could be associated with disturbing dreams in the preclinical stages of dementia more broadly. If so, age and sex may play a role, as postmortem Lewy bodies are less prevalent in individuals with dementia onset at 70–79 years compared to those aged 60–69 or 80 and above and more prevalent in men than women.^15^

A second possibility involves the brain's salience network, primarily comprising anterior regions of the insular and cingulate cortices.^16^ Overactivation of the salience network is associated with exaggerated responsivity, anxiety, and panic.^17^ While usually deactivated during rapid eye movement (REM) sleep,^18^ evidence suggests the salience network is active during REM sleep in nightmare experiencers,^19^ consistent with higher insular activity during REM sleep being associated with fear in dreams.^20^ The salience network shows hyperconnectivity among cognitively normal older adults positive for beta‐amyloid, but only when tau levels are low.^21^ A hyperconnected and overactive salience network may enhance sensitivity to stimuli and potential threats associated with post‐traumatic stress disorder (PTSD),^17^ a condition characterized by frequent nightmares.^22^ It thus seems possible that hyperconnectivity and greater activity of the salience network could promote disturbing dreams in cognitively normal individuals with early beta‐amyloid pathology.

A third possible link between disturbing dreams and future dementia involves stress. Associations between stress and nightmares were considered in Hartmann's seminal book on nightmares,^23^ with later studies supporting an association between daily life stress and nightmares.^24^, ^25^ Chronic stress leads to an attenuated range of cortisol levels, including a reduced peak of the cortisol awakening response (CAR).^26^ Some studies have found that a larger CAR was associated with better current and future cognition.^26^, ^27^ Frequent nightmares were found to be associated with a reduced CAR,^28^ and disturbing dreams are thus potentially a marker of altered cortisol functioning that is, in turn, associated with future dementia. There may be parallels in PTSD involving a high prevalence of nightmares^22^ and being a risk factor for dementia.^29^ However, nightmares have a bidirectional association with stress, and disturbing dreams may not simply be a response to stress but also a contributor.^24^ This suggests that disturbing dreams could also be an indirect contributor to dementia, via stress and concomitant effects such as reduced sleep hours.^30^ It is also possible that elevated stress levels could be a prodromal symptom of dementia,^31^ and that disturbing dreams are a result of this.

While there are numerous potential mechanisms, the evidence for disturbing dreams being associated with a greater risk of future dementia is currently limited. Otaiku's^9^ reported association between disturbing dreams and dementia was based on predominantly white US participants, mostly aged 79–89 years, and for whom a positive incident dementia status required an independently obtained professional diagnosis between baseline and follow‐up. The aim of the current study is to address some of these generalizability and methodological issues to clarify if disturbing dreams are associated with dementia in community‐based older adults. Our study used a large and diverse sample comprising six cohorts from Asia, Europe, and South America that included participants aged 60–89 years. Further, dementia classifications were determined at multiple times during follow‐up according to either consensus diagnoses or cognitive test results. We investigated potential associations between disturbing dreams and not only all‐cause dementia but also AD. We also aimed to investigate associations between disturbing dreams and DLB and PDD but were limited to a descriptive account of this because incident cases were rare.

## Methods

### Participating cohorts

All six contributing cohort studies were population‐based and members of Cohort Studies of Memory in an International Consortium (COSMIC).^32^ Table 1 shows study details (including abbreviations). Among participants with baseline disturbing dream data and at least one follow‐up assessment, we excluded those with baseline dementia or missing dementia data, as well as anyone with baseline PD given abnormally high disturbing dream frequency.^33^ We also excluded 12 Korean War veterans from KLOSCAD, given effects of war exposure on disturbing dream frequency.^34^ This left us with an overall sample of 10,238 individuals (Table S1 shows the study‐level exclusion numbers). The project was approved by the University of New South Wales Human Research Ethics Committee (HC12446, HC17292, and HC220222) and the contributing studies had prior ethics approval (Table S2).

**Table 1:** Contributing cohort studies

Study	Abbreviation	Location	Baseline year(s)	Maximum waves (*n*); follow‐up (years)	References
Bambui Cohort Study of Ageing	BCSA	Bambui, Brazil	1997	16; 15.0	Costa *et al*.^35^
Enquête de Santé Psychologique‐Risques, Incidence et Traitement	ESPRIT	Montpellier, France	1999–2001	7; 17.2	Ritchie *et al*.^36^
Invecchiamento Cerebrale in Abbiategrasso	InveCe.Ab	Abbiategrasso, Italy	2010	4; 9.1	Guaita *et al*.^37^
Korean Longitudinal Study on Cognitive Aging and Dementia	KLOSCAD	South Korea (nationwide)	2010–2012	4; 7.9	Han *et al*.^38^
Shanghai Aging Study	SAS	Shanghai, China	2010–2011	2; 7.3	Ding *et al*.^39^
Taiwan Initiatives for Geriatric Epidemiological Research	TIGER	Taipei, Taiwan	2011–2013	3; 5.7	Hsieh *et al*.^40^

### Disturbing dreams

Two studies asked participants a yes/no question about nightmares, if waking up because of nightmares was a problem in the last month (BCSA) or if they have nightmares (ESPRIT). The psychometric properties of such questions have received little attention, though one study found that responses to the statement ‘I have nightmares frequently (true/false)’ were moderately correlated with scores on the Nightmare Distress Questionnaire.^41^ The other four studies (InveCe.Ab, KLOSCAD, SAS, and TIGER) administered the Pittsburgh Sleep Quality Index (PSQI) item on trouble sleeping because of bad dreams during the past month,^42^ with four response options from ‘Not during the past month’ to ‘Three or more times a week’. While the psychometric properties of this particular question do not appear to have been determined, the PSQI overall shows strong reliability and validity.^43^ For analyses involving all studies, we created a binary variable by collapsing all PSQI item response options other than ‘Not during the past month’ into one category indicating at least one bad dream during the past month. Past research found the strongest associations between disturbing dreams and dementia risk for individuals reporting at least one disturbing dream per week.^4^, ^9^ Given this, we also investigated effects in terms of disturbing dream frequency using the PSQI item data, collapsing the upper two response categories into ‘At least once a week’ (≥1/week) and the lower two categories into ‘Less than once a week’ (<1/week). As a supplementary investigation, we repeated these analyses using three categories for disturbing dream frequency in the past month: less than monthly (‘Not during the past month’), monthly (‘Less than once a week’), and weekly (‘At least once a week’ or ‘Three or more times a week’). This is because it might be expected that differences in dementia risk are greatest between less than monthly and weekly disturbing dreams.

### All‐cause dementia and AD

Incident dementia was assessed in all waves of each study. Four studies (ESPRIT, InveCe.Ab, KLOSCAD, and SAS) diagnosed all‐cause dementia as per Diagnostic and Statistical Manual of Mental Disorders, Fourth Edition^44^ criteria; they also diagnosed AD using criteria of the National Institute of Neurological and Communicative Disorders and Stroke and the Alzheimer's Disease and Related Disorders Association.^45^ BCSA classified all‐cause dementia using a Mini‐Mental State Examination (MMSE)^46^ score <14, appropriate for Brazilian populations with low schooling.^47^ TIGER classified dementia as a Montreal Cognitive Assessment (MoCA; Taiwanese version) score <22,^48^ self‐report of dementia, or the use of AD medication. We assigned the dementia/AD onset date as mid‐way between the date when first diagnosed/classified and the previous assessment date. Three studies (ESPRIT, KLOSCAD, and SAS) diagnosed DLB and PDD, as per consensus guidelines.^49^

### Covariates

We performed analyses adjusted for age and sex with the full sample from all six studies that contributed data. We also performed fully adjusted analyses using three studies (InveCe.Ab, KLOSCAD, and TIGER) that had data for additional covariates. These covariates were chosen for their potential associations with disturbing dreams and/or dementia/AD.^9^, ^50^ Harmonization was needed, as per previous COSMIC studies^51^ (for procedures see Tables S3–S7). Demographic covariates beyond age and sex were years of education, marital status (married, widow/er, other), and income (a three‐level ordinal variable). Most of the other covariates were coded as yes/no: sleep onset insomnia, sleep maintenance insomnia, extreme sleep hours (<5 or >10 h), daytime sleepiness, snoring, medications for sleep or that may influence disturbing dream frequency, history of depression, state depression, anxiety, hypertension, diabetes, cardiovascular disease, history of stroke, physical inactivity, current alcohol drinker, current smoker, apolipoprotein E (APOE) gene ε4 allele carriage (having at least one ε4 allele). Additional covariates were MMSE score (standardized across studies as outlined in Supporting information Methods), body mass index, and self‐rated health (poor, fair, good/excellent). InveCe.Ab did not have baseline sleep hour data, so for this variable and study, we used values from the nearest assessment with these data, around 4 years later.

From 6,359 individuals across the three studies, 1,066 (16.8%) did not have complete covariate data (Table S1). We compared the characteristics of individuals with complete covariate data to those without these data. There were many significant differences, suggesting that covariates were not missing at random and that the group with incomplete covariates comprised a distinct subgroup with poorer physical and mental health (Table S8). Given this, we conducted complete case fully adjusted analyses, but for comparison also conducted analyses retaining individuals without complete covariates that only adjusted for age and sex.

### Statistical analysis

We formed three age groups: 60–69, 70–79, and 80–89 years for age stratified analyses. This was done for two reasons: (i) facilitation of comparison with prior research^9^ and (ii) because of potential age effects associated with a Lewy body contribution to disturbing dreams, given reported differences in the prevalence of Lewy bodies across age of dementia onset groups.^15^ Sex‐stratified analyses were performed for related reasons, with associations between disturbing dreams and incident dementia previously found only in men,^9^ and men having a greater prevalence of Lewy bodies than women.^15^ Between‐group differences in disturbing dream prevalence and covariates were investigated using chi‐square or independent sample *t*‐tests. Mixed‐effect Cox proportional‐hazard regression models were performed to assess the risk of follow‐up dementia associated with having disturbing dreams, provided as adjusted hazard ratios (HRs) and 95% confidence intervals (CIs). Time‐to‐diagnosis was included as the underlying time scale. The Cox models were performed for all participants, as well as stratified by age and separately by sex. We conducted a main set of analyses using data for all six contributing studies, and that adjusted for age group and sex (unless the focus of a stratified analysis). These were repeated in sensitivity analyses that either featured only the four studies with dementia diagnosis data (rather than scale cut‐off scores) or excluded individuals with low cognition (MMSE *z*‐score <−1.5), given a report of stronger associations between disturbing dreams and incident dementia when excluding mild cognitive impairment cases.^9^ Using the three studies with full covariate data, we also conducted sets of analyses that were either fully adjusted or only adjusted for age group and sex (as per the main analyses). Models were performed separately for all‐cause dementia and AD as outcomes. Table S9 shows how studies were included in the different analyses based on data availability. InveCe.Ab was not included in age group analyses other than 70–79 years as all participants were aged 70–75 years at baseline. All Cox analyses included a random effect of study and were conducted in R studio (version 4.4.2) using the ‘coxme’ package.^52^ Statistical significance was determined by a two‐sided *P*‐value <0.05. To make allowance for multiple comparisons in the age‐stratified analyses, *P*‐values were Bonferroni‐adjusted by multiplying the original values by three (reflecting three age groups), and for sex‐stratified analyses, by multiplying by two (reflecting two sex groups). As the supplementary investigation using three disturbing dream frequency categories featured twice the number of comparisons, we Bonferroni‐adjusted by multiplying the original *P*‐values by six and four within each age and sex group, respectively. Original *P*‐values are presented in tables, while Bonferroni‐adjusted values are cited when discussing statistical significance in the text.

## Results

### Sample

A total of 10,238 participants had disturbing dream and incident dementia data. Their mean baseline age was 70.5 (SD 6.0) years, 42.5% were men, and they had a mean of 8.2 (SD 5.1) years of education (Table S10 shows the study‐level demographic characteristics).

### Disturbing dreams

The prevalence of any disturbing dreams across all studies was 24.2%. For 6,940 participants with frequency data, 6.6% had disturbing dreams ≥1/week. Table 2 shows the sample size and disturbing dream prevalence for each age and sex group. The prevalence of any disturbing dreams did not differ by age (*Χ* ^2^ = 4.69, *P* > 0.05) but the proportion reporting disturbing dreams ≥1/week increased with age (*Χ* ^2^ = 14.77, *P* < 0.01). Compared to men, women had a higher prevalence of any disturbing dreams (*Χ* ^2^ = 16.45, *P* < 0.001) and a higher proportion had disturbing dreams ≥1/week (*Χ* ^2^ = 9.71, *P* < 0.01). The prevalence of disturbing dreams ranged from 12.1% to 28.7% across the studies (Table S11).

**Table 2:** Sample size and disturbing dream prevalence for each age and sex group

Group	Any disturbing dreams, *n* (%)^†^		Disturbing dream frequency, *n* (%)^‡^		
	No	Yes	<1/week	≥1/week	
Overall	7761 (75.8)	2477 (24.2)	6479 (93.4)	461 (6.6)	
Age, years					
60–69	3381 (76.1)	1064 (23.9)	2820 (94.3)	171 (5.7)	
70–79	3740 (75.1)	1238 (24.9)	3231 (93.1)	241 (6.9)	
80–89	640 (78.5)	175 (21.5)	428 (89.7)	49 (10.3)	
Sex					
Female	4372 (74.3)	1510 (25.7)	3607 (92.5)	291 (7.5)	
Male	3389 (77.8)	967 (22.2)	2872 (94.4)	170 (5.6)	

^†^ All six studies had data for any disturbing dreams (*n* = 10,238).

^‡^ Four studies had disturbing dream frequency data (*n* = 6940).

For the 5,293 individuals with complete covariate data, Table S12 shows the prevalence or mean value of each covariate according to disturbing dream status (no/yes). All sleep‐related (insomnia, extreme sleep hours, sleepiness, snoring, and medications) and mental health (depression and anxiety) factors were associated with a higher level of disturbing dreams. Having disturbing dreams was more likely for individuals with poor health or who were current alcohol drinkers.

### All‐cause dementia and AD incidence

The maximum and mean follow‐up duration across studies was 17.2 years and 6.5 (SD 3.9) years, respectively. There were 726 (7.1%) incident dementia cases, with a mean onset time of 4.9 (SD 3.5) years. Across the four studies with AD data, there were 357 (4.3%) incident AD cases, with a mean onset time of 4.3 (SD 3.5) years. Dementia and AD incidence was 10.8 and 5.3 per 1000 person‐years, respectively. The percentage of incident dementia and AD cases increased with age and was greater for women than for men (Table S13); it also varied across studies (Table S14).

### Associations between disturbing dreams and incident all‐cause dementia and AD

#### Any disturbing dreams

In the main analyses featuring all six contributing studies, there were no significant associations between having disturbing dreams and incident all‐cause dementia or AD overall or within any age or sex group (Table S15). No significant effects emerged when limiting the analyses to the four studies with dementia diagnoses (ESPRIT, InveCe.Ab, KLOSCAD, and SAS) or excluding individuals with lower cognition (results not shown).

Table 3 shows the results of fully adjusted analyses featuring three studies (InveCe.Ab, KLOSCAD, and TIGER). There was a significant association between disturbing dreams and all‐cause dementia among individuals aged 60–69 years (Bonferroni‐adjusted *P* = 0.042), and disturbing dreams tended to be associated with increased chances of AD among men (Bonferroni‐adjusted *P* = 0.094). Analyses including the participants missing covariates and only adjusting for age and sex also found that disturbing dreams were significantly associated with greater chances of all‐cause dementia among individuals aged 60–69 years (HR 2.91, 95% CI 1.35–6.29, Bonferroni‐adjusted *P* = 0.021; Table S16). There was an additional significant association between disturbing dreams and AD in this age group (HR 3.39, 95% CI 1.27–9.03, Bonferroni‐adjusted *P* = 0.045).

**Table 3:** Risk of incident all‐cause and Alzheimer dementia associated with any disturbing dreams in the past month: fully adjusted models with individuals from InveCe.Ab, KLOSCAD, and TIGER^†^

Group	Disturbing dreams	All‐cause dementia^‡^			Alzheimer dementia^§^		
		*n*/*n*[event]	HR (95% CI)	*P*‐value	*n*/*n*[event]	HR (95% CI)	*P*‐value
All	No	4040/186	1.00 (ref)		3705/111	1.00 (ref)	
	Yes	1253/60	1.04 (0.76–1.42)	0.803	1196/38	1.21 (0.82–1.79)	0.340
60–69 years	No	1849/9	1.00 (ref)		1819/4	1.00 (ref)	
	Yes	561/10	3.93 (1.32–11.67)	0.014*	552/6	4.96 (0.97–25.38)	0.054
70–79 years	No	1978/141	1.00 (ref)		1726/83	1.00 (ref)	
	Yes	631/38	0.92 (0.63–1.35)	0.681	590/23	0.97 (0.59–1.58)	0.894
80–89 years	No	213/36	1.00 (ref)		160/24	1.00 (ref)	
	Yes	61/12	1.05 (0.47–2.33)	0.909	54/9	1.24 (0.45–3.38)	0.671
Women	No	2224/115	1.00 (ref)		2044/80	1.00 (ref)	
	Yes	687/31	0.87 (0.57–1.33)	0.514	659/23	0.90 (0.55–1.48)	0.672
Men	No	1816/71	1.00 (ref)		1661/31	1.00 (ref)	
	Yes	566/29	1.27 (0.79–2.05)	0.330	537/15	2.03 (1.01–4.09)	0.047

* *P* < 0.05, following adjustment for multiple comparisons within the age and sex groups.

^†^ Models are adjusted for age, sex, years of education, marital status, income, sleep onset insomnia, sleep maintenance insomnia, extreme sleep hours, daytime sleepiness, snoring, medications for sleep or that may influence disturbing dream frequency, history of depression, state depression, anxiety, hypertension, diabetes, cardiovascular disease, history of stroke, physical inactivity, current alcohol drinker, current smoker, APOE ε4 allele carriage, MMSE score, body mass index, and self‐rated health.

^‡^ Number of studies in the models = 3 (InveCe.Ab, KLOSCAD, TIGER), except for 2 (KLOSCAD, TIGER) in the 60–69‐year and 80–89‐year group models.

^§^ Number of studies in the models = 2 (InveCe.Ab, KLOSCAD), except for 1 (KLOSCAD) in the 60–69‐year and 80–89‐year group models.

CI, confidence interval; HR, hazard ratio; ref, reference group.

#### One or more disturbing dreams per week

For the four studies with disturbing dream frequency data (InveCe.Ab, KLOSCAD, SAS, and TIGER), the main analyses found that disturbing dreams ≥1/week tended to be associated with a greater risk of all‐cause dementia over all individuals (HR 1.36, 95% CI 0.97–1.90, *P* = 0.074), and with greater chances of AD among individuals aged 60–69 years (HR 3.95, 95% CI 1.13–13.85, Bonferroni‐adjusted *P* = 0.096) (Table S17). No significant effects emerged when limiting the analyses to the three studies with dementia diagnoses (InveCe.Ab, KLOSCAD, and SAS), but excluding individuals with lower cognition yielded a significant association between disturbing dreams ≥1/week and all‐cause dementia among individuals aged 60–69 years (HR 5.04, 95% CI 1.37–18.48, Bonferroni‐adjusted *P* = 0.044; other results not shown).

The fully adjusted analyses featuring three studies (InveCe.Ab, KLOSCAD, and TIGER) found a significant association between disturbing dreams ≥1/week and greater chances of AD among men (adjusted *P* = 0.012), and a trend for disturbing dreams ≥1/week to be associated with greater chances of AD across all participants (*P* = 0.056) (Table 4). Analyses including the participants missing covariates and only adjusting for age and sex replicated these findings (for men HR 2.43, 95% CI 1.20–4.94, Bonferroni‐adjusted *P* = 0.042; for all participants HR 1.50, 95% CI 0.98–2.30, *P* = 0.063; Table S18); they also found a significant association over all participants for all‐cause dementia (HR 1.43, 95% CI 1.01–2.03, *P* = 0.045) and a trend for AD among individuals aged 60–69 years (HR 4.06, 95% CI 1.15–14.34, Bonferroni‐adjusted *P* = 0.090).

**Table 4:** Risk of incident all‐cause and Alzheimer dementia associated with disturbing dreams ≥1/week in the past month: Fully adjusted models with individuals from InveCe.Ab, KLOSCAD, and TIGER^†^

Group	Disturbing dreams	All‐cause dementia^‡^			Alzheimer dementia^§^		
		*n*/*n*[event]	HR (95% CI)	*P*‐value	*n*/*n*[event]	HR (95% CI)	*P*‐value
All	<1/week	4956/223	1.00 (ref)		4585/132	1.00 (ref)	
	≥1/week	337/23	1.21 (0.76–1.92)	0.417	316/17	1.70 (0.99–2.93)	0.056
60–69 years	<1/week	2275/17	1.00 (ref)		2240/9	1.00 (ref)	
	≥1/week	135/2	0.99 (0.18–5.33)	0.987	131/1	0.62 (0.05–7.70)	0.714
70–79 years	<1/week	2435/163	1.00 (ref)		2155/94	1.00 (ref)	
	≥1/week	174/16	1.45 (0.84–2.49)	0.182	161/12	1.97 (1.04–3.73)	0.037
80–89 years	<1/week	246/43	1.00 (ref)		190/29	1.00 (ref)	
	≥1/week	28/5	0.88 (0.31–2.51)	0.806	24/4	1.35 (0.36–5.02)	0.655
Women	<1/week	2713/133	1.00 (ref)		2515/93	1.00 (ref)	
	≥1/week	198/13	1.15 (0.62–2.12)	0.653	188/10	1.23 (0.61–2.48)	0.566
Men	<1/week	2243/90	1.00 (ref)		2070/39	1.00 (ref)	
	≥1/week	139/10	1.80 (0.88–3.68)	0.651	128/7	3.59 (1.44–8.96)	0.006*

* *P* < 0.05, following adjustment for multiple comparisons within the age and sex groups.

^†^ Models are adjusted for age group, sex, years of education, marital status, income, sleep onset insomnia, sleep maintenance insomnia, extreme sleep hours, daytime sleepiness, snoring, medications for sleep or that may influence disturbing dream frequency, history of depression, state depression, anxiety, hypertension, diabetes, cardiovascular disease, history of stroke, physical inactivity, current alcohol drinker, current smoker, APOE ε4 allele carriage, MMSE score, body mass index, and self‐rated health.

^‡^ Number of studies in the models = 3 (InveCe.Ab, KLOSCAD, TIGER), except for 2 (KLOSCAD, TIGER) in the 60–69‐year and 80–89‐year group models.

^§^ Number of studies in the models = 2 (InveCe.Ab, KLOSCAD), except for 1 (KLOSCAD) in the 60–69‐year and 80–89‐year group models.

CI, confidence interval; HR, hazard ratio; ref, reference group.

There were similar results for weekly (≥1/week) disturbing dreams in the supplementary investigation using three frequency categories, with less than monthly as the reference group. Men in the fully adjusted analysis featuring three studies (InveCe.Ab, KLOSCAD, and TIGER) showed a greater risk of AD (HR 4.00, 95% CI 1.55–10.31, Bonferroni‐adjusted *P* = 0.016; Table S19). When the participants from these studies missing covariates were included and the analyses only adjusted for age and sex, there were trends for weekly disturbing dreams to be associated with greater risk of dementia among all participants (HR 1.39, 95% CI 0.98–1.98, *P* = 0.071) and AD among men (HR 2.38, 95% CI 1.16–4.88, Bonferroni‐adjusted *P* = 0.072; Table S20). There were no effects for weekly disturbing dreams beyond those observed in the initial analyses comparing ≥1/week against <1/week. However, we note that in the main analysis with four studies (InveCe.Ab, KLOSCAD, SAS, and TIGER), and generally in the other analyses, that HRs were greater with weekly than monthly disturbing dreams. This includes when monthly disturbing dreams were significantly associated with greater risk of dementia in the 60–69 years group (HR 3.15, 95% CI 1.41–7.01, Bonferroni‐adjusted *P* = 0.030, Table S21). Conversely, among all participants, individuals aged 70–79 years, and women, the HRs with monthly disturbing dreams were consistently (though nonsignificantly) less than one.

### Descriptive account of incident DLB and PDD

Across the four studies with dementia type data (ESPRIT, InveCe.Ab, KLOSCAD, and SAS) there were 16 and 5 incident DLB and PDD cases, respectively. DLB/PDD accounted for 4.8% (10/207) and 3.2% (11/350) of all dementia among men and women, respectively, and for 11.7% (7/60), 3.2% (12/375), and 1.6% (2/122) of all dementia among individuals aged 60–69, 70–79, and 80–89 years, respectively. The baseline prevalence of disturbing dreams among incident DLB/PDD cases was 47.6%, compared to 24.1% and 22.4% among incident all‐cause dementia and AD cases, respectively. The prevalence among individuals who remained dementia free was 24.1%. Similar patterns were found for the two studies with dementia type data included in the fully adjusted analyses (InveCe.Ab and KLOSCAD), though the difference in DLB/PDD incidence between men (5.8%) and women (1.7%) was more extreme (see Supporting information results for full details).

## Discussion

Using data from diverse international cohort studies, we found some evidence suggesting that disturbing dreams are associated with increased incidence of all‐cause dementia and AD. This was with a subset of the contributing studies and primarily for all‐cause dementia among individuals aged 60–69 years having any disturbing dreams and for AD among men having at least one disturbing dream per week. These results are broadly consistent with previous findings that disturbing dreams were associated with cognitive decline among middle‐aged adults and with incident all‐cause dementia in older adults.^9^ We extend these results by using data from diverse international regions and by finding associations between disturbing dreams and AD specifically, rather than only all‐cause dementia.

The overall 24% prevalence of disturbing dreams we found for adults aged 60–89 years fits well with the 27% for adults aged 35–64 years and 20% for adults aged 79 years or more found by Otaiku,^9^ who used the same scale (PSQI item) as most of our contributing studies. While the prevalence of any disturbing dreams did not differ by age among our participants, the proportion reporting disturbing dreams ≥1/week increased with age. Similar age trends for older adults have been previously reported, but there were no individuals older than 74 years in that study.^34^ We also found that women had a higher prevalence of disturbing dreams than men. Women are reported to have a higher prevalence of disturbing dreams than men for most of the lifespan,^53^ a disparity some suggest is maintained at all ages^10^ and others find to recede in later life.^34^, ^54^ Other factors associated with a greater likelihood of having disturbing dreams overlapped considerably between the previous and current studies, including lower cognition, insomnia, extreme sleep hours, daytime sleepiness, snoring, depression, anxiety, use of sleep or other potentially influential medications, and poor self‐rated health. Importantly, disturbing dreams showed associations with incident all‐cause dementia and AD even when controlling for these factors.

While we found significant associations between disturbing dreams and AD only among men, the HR was higher among men than women in all analyses with all‐cause dementia as the outcome. This suggests that the associations between disturbing dreams and dementia apply to men but not women, consistent with the sex effect for all‐cause dementia reported by Otaiku.^9^ However, while Otaiku^9^ found an association between disturbing dreams and dementia using a sample mostly aged 79–89 years, we did not find a similar association for our 80–89‐year‐old group. A possible influence on this difference is Otaiku's^9^ study having only one follow‐up assessment, while we included data from multiple waves, mitigating the effects of loss to follow‐up. Another is the different means of identifying incident dementia, an independent professional diagnosis in Otaiku's study *versus* our use of consensus diagnoses and screening test cut‐off scores (noting that we conducted sensitivity analyses that included data only from consensus diagnoses). Further, our 80–89‐year‐old group had a similar level of disturbing dreams to Otaiku's^9^ sample (23% vs 20%), but more than double the proportion with incident dementia (20% vs 9%; also when restricting our sample to the maximum 7 years of follow‐up in Otaiku's sample). Older individuals with lower general dream recall are reported to be at greater risk of future dementia.^55^ It is thus possible that associations between disturbing dreams and future dementia are less likely to be found in samples with more individuals at risk of dementia who do not report disturbing dreams because of reduced overall dream recall. This may also explain our finding of a significant association between disturbing dreams ≥1/week and all‐cause dementia for the 60–69‐year group that emerged when excluding individuals with poor cognition.

How many individuals reporting less than monthly disturbing dreams had low general dream recall associated with greater dementia risk is unknown, but their presence in this reference group may have contributed to the HRs with monthly disturbing dreams often being (nonsignificantly) less than one. Conversely, the HRs with weekly disturbing dreams were preponderantly greater than one and the HRs with monthly disturbing dreams. Correspondingly, we found significant associations between disturbing dreams and AD for men reporting weekly disturbing dreams, but not for men reporting any or monthly disturbing dreams. A similar pattern was not clearly found among the 60–69‐year group, in whom a greater risk of all‐cause dementia was significantly associated with any disturbing dreams, but not with weekly disturbing dreams after *P*‐value adjustment for multiple comparisons. Generally, however, our results are consistent with Otaiku's^9^ conclusion that ‘a higher frequency of distressing dreams may represent a more advanced stage of neurodegeneration’ (p. 11). Higher disturbing dream frequency and lower dream recall thus both seem associated with greater dementia risk, but their mutual exclusivity suggests that they may stem from different neurodegenerative processes.

Four possible mechanisms underlying associations between disturbing dreams and future dementia were outlined earlier. In brief, these involve a neurocognitive model of nightmares, Lewy bodies, the salience network, and stress. Our study was neither intended nor had the data to directly test any of these, but the results show some consistency with potential Lewy body involvement. Lewy body disorders show a particularly strong association with disturbing dreams,^12^ and Lewy bodies are a common co‐pathology in AD.^14^ Concomitant Lewy bodies and AD pathology exacerbate cognitive decline^56^ and reduce the dementia onset age.^14^, ^15^ Further, Lewy bodies are more common in men than women, including when concomitant with AD pathology.^15^ These age and sex differences in Lewy body occurrence could help explain our findings of disturbing dreams being associated with all‐cause dementia only among individuals aged 60–69 years at baseline, and with AD among men but not women. While we cannot know how many incident AD cases had concomitant Lewy bodies in our study, incident DLB and PDD were more frequent in participants aged 60–69 years at baseline than in participants aged 70–89 years at baseline, and in men than women. Further, the baseline prevalence of disturbing dreams was greater among those who developed DLB or PDD than those who developed all‐cause dementia, consistent with previous findings.^7^, ^12^ How Lewy body pathology may influence disturbing dream frequency is unknown.

No significant associations between any disturbing dreams and dementia were found in analyses that included the studies BCSA and ESPRIT. These studies asked questions about nightmares while all other studies administered the PSQI item asking about ‘bad dreams’. Further, unlike all other studies, ESPRIT's disturbing dream question did not specify that responses should refer to the previous month, possibly leading to an inflated amount of individuals reporting that they have disturbing dreams. BCSA and ESPRIT also differed by having longer follow‐up durations than the other studies, and accordingly a larger overall proportion of individuals who developed dementia. However, an exploratory analysis excluding individuals from those studies with follow‐up durations exceeding the maximum duration of the other studies suggests this was not an influence (results not shown). We note that for the analyses involving disturbing dreams ≥1/week, BCSA and ESPRIT did not collect frequency data and were thus never included.

REM sleep behavior disorder (RBD) is unlikely to have influenced the associations between disturbing dreams and dementia that we found. RBD is a prodrome of Lewy body disease in which a lack of muscle atonia can lead to dream enactment behaviors during REM sleep.^57^ Some studies report a higher frequency of nightmares in RBD patients than in controls,^58^ but others do not.^59^ While RBD is caused by Lewy bodies in the brainstem,^60^ individuals later diagnosed with AD accrue Lewy bodies predominantly in the amygdala rather than the brainstem.^14^ Also, RBD is found in only around 1% of the general population.^61^ The largest cohort in our study, KLOSCAD, had only six participants who reported RBD at baseline, three of whom were excluded from our analyses for having baseline dementia (one case) or PD (two cases). Of the other three, two reported disturbing dreams and none developed dementia.

Strengths of our study include having data from various international cohort studies, giving a large overall sample size and enhancing the robustness of our results. We investigated associations between disturbing dreams and not only all‐cause dementia, but also AD. Our analyses were adjusted for many factors associated with disturbing dreams, giving us more confidence that our findings represent independent associations between disturbing dreams and the risk of all‐cause dementia and AD. However, we did not have data to adjust for past or current experience of stress or for PTSD. We were also limited by the relatively small sample size for our oldest age group (80–89 years), possibly reducing the chances of finding statistically significant associations. For two studies without dementia diagnoses (BCSA and TIGER), we classified dementia using cognitive screening test scores. This compromise approach has been used in COSMIC studies to increase inclusion and diversity, though at the expense of diagnostic accuracy and potential dilution of the true effects to be found.^62^ However, sensitivity analyses excluding these studies did not alter the results, and we note that these two studies did not feature in our analyses with AD as the outcome. Our fully adjusted analyses omitted individuals missing complete covariate data, and who had poorer physical and mental health than included individuals. However, analyses only adjusting for age and sex that included the individuals missing complete covariate data produced results largely the same as the fully adjusted analyses. An exception was a significant overall effect for disturbing dreams ≥1/week and all‐cause dementia among all participants not being found in the fully adjusted analyses, though noting that a significant association with AD among men was found in both.

Also to consider when interpreting our results is a report that older adults alive during World War II had higher rates of nightmares, particularly war veterans.^34^ We had data to exclude war veterans only from KLOSCAD, though note that all KLOSCAD participants were alive during the Korean War (1950–1953; Table S22 shows the birth year ranges of the studies). Similarly, most SAS participants were teenagers or young adults when the Chinese Civil War ended in 1949, and ESPRIT participants spent part of their child‐ or adulthood during World War II (1939–1945). We also know that 13% of ESPRIT participants were repatriated from Algeria to France in 1962 because of the Algerian War, with 7% of that group experiencing a war‐related traumatic event.^63^ It is unclear to what extent these exposures influenced the prevalence of disturbing dreams decades later. However, we note that ESPRIT had a lower disturbing dream prevalence than BCSA and InveCe.Ab. Participants in InveCe.Ab were children in Italy during World War II, and while BCSA participants were alive at the time, Brazil had minimal involvement in the war. Further, the prevalence of disturbing dreams was similar across all studies except TIGER, and most cohorts born in the early or mid‐20th century have been exposed to global or national conditions that may affect disturbing dream prevalence, which could include economic recession rather than war.^34^

## Conclusions

Using data from diverse international cohorts, we found that disturbing dreams were associated with incident all‐cause dementia among individuals aged 60–69 years and with AD among men. The mechanisms potentially underlying these associations remain to be clarified.

## Author contributions

Conceptualization was performed by D.M.L. Methodology was developed by D.M.L. and A.S.V. Formal analysis was performed by A.S.V. Investigation was done by E.C.‐C., K.R., E.R., A.D., K.W.K., J.W.H., D.J.O., D.D., Y.‐C.C., and J.‐H.C. Resources were provided by E.C.‐C., K.R., E.R., A.D., K.W.K., J.W.H., D.J.O., D.D., Y.‐C.C., and J.‐H.C. Data Curation was performed by D.M.L. Original draft was written by D.M.L. Review and editing were done by D.M.L., A.S.V., E.C.‐C., S.L.B., M.F.L.‐C., K.R., E.R., A.D., M.R., K.W.K., J.W.H., D.J.O., D.D., QZ, XZ, Y.‐C.C., J.‐H.C., and P.S.S. Project administration was done by D.M.L. and P.S.S. Funding acquisition was done by P.S.S.

## Disclosure statement

The authors have no conflicts of interest to declare.

## Supporting information


**Data S1.** Supporting information.

## Data Availability

Data were provided by the contributing studies to COSMIC on the understanding and proviso that the relevant study leaders be contacted for further use of their data and additional formal data sharing agreements be made. Researchers can apply to use COSMIC data via Dementias Platform Australia (DPAU): https://portal.dementiasplatform.com.au/data-application.
