# Supplementary material for: Disturbing dreams and dementia incidence across diverse cohort studies: A COSMIC collaboration study
Source: Psychiatry Clin Neurosci. 2026 Mar 17;80(7):566–74. doi: 10.1111/pcn.70046 (PMC13332577; doi:10.1111/pcn.70046)
Supplement: Supplementary file 1 — Data S1. Supporting information. [file PCN-80-566-s001.docx]

**SUPPLEMENTARY MATERIAL for Lipnicki et al. Disturbing dreams and dementia incidence across diverse cohort studies: A COSMIC collaboration study**

Contents

[**Supplementary methods:** Standardization of outcome scores 3](#_Toc218859366)

[**Supplementary results:** Incident DLB and PDD in the two studies included in the fully adjusted analyses 4](#_Toc218859367)

[**Supplementary Table 1.** Exclusions and missingness 5](#_Toc218859368)

[**Supplementary Table 2.** Contributing study ethics approvals 6](#_Toc218859369)

[**Supplementary Table 3.** Harmonisation of income 7](#_Toc218859370)

[**Supplementary Table 4.** Harmonisation of depression and anxiety 8](#_Toc218859371)

[**Supplementary Table 5.** Harmonisation of insomnia, snoring, daytime sleepiness, medications for sleep or influencing disturbing dream frequency 9](#_Toc218859372)

[**Supplementary Table 6.** Harmonisation of cardiovascular disease, hypertension, and diabetes 10](#_Toc218859373)

[**Supplementary Table 7.** Harmonisation of physical activity, self-reported health 11](#_Toc218859374)

[**Supplementary Table 8.** Characteristics of individuals either included or not included in the fully adjusted analyses featuring data from three studies 12](#_Toc218859375)

[**Supplementary Table 9.** Availability of disturbing dream, dementia/AD, and complete covariate data across studies 14](#_Toc218859376)

[**Supplementary Table 10.** Baseline demographic characteristics of the included participants for each cohort study 15](#_Toc218859377)

[**Supplementary Table 12.** Covariate data for disturbing dream groups (no/yes) 17](#_Toc218859378)

[**Supplementary Table 13.** Follow-up durations, incidence of dementia and AD, and time from baseline to incident all-cause dementia or AD 19](#_Toc218859379)

[**Supplementary Table 14.** Follow-up durations and dementia outcomes for the studies 20](#_Toc218859380)

[**Supplementary Table 15.** Risk of incident all-cause and Alzheimer dementia associated with any disturbing dreams: all individuals from BCSA, ESPRIT, Invece.Ab, KLOSCAD, SAS, and TIGER 21](#_Toc218859381)

[**Supplementary Table 16.** Risk of incident all-cause and Alzheimer dementia associated with any disturbing dreams in the past month: all individuals from Invece.Ab, KLOSCAD, and TIGER 22](#_Toc218859382)

[**Supplementary Table 17.** Risk of incident all-cause and Alzheimer dementia associated with disturbing dreams ≥1/wk in the past month: all individuals from Invece.Ab, KLOSCAD, SAS, and TIGER 23](#_Toc218859383)

[**Supplementary Table 18.** Risk of incident all-cause and Alzheimer dementia associated with disturbing dreams ≥1/wk in the past month: all individuals from Invece.Ab, KLOSCAD, and TIGER 24](#_Toc218859384)

[**Supplementary Table 19.** Risk of incident all-cause and Alzheimer dementia associated with disturbing dream frequency in the past month: fully adjusted models with individuals from Invece.Ab, KLOSCAD, and TIGER 25](#_Toc218859385)

[**Supplementary Table 20.** Risk of incident all-cause and Alzheimer dementia associated with disturbing dream frequency in the past month: all individuals from Invece.Ab, KLOSCAD, and TIGER 26](#_Toc218859386)

[**Supplementary Table 21.** Risk of incident all-cause and Alzheimer dementia associated with disturbing dream frequency in the past month: all individuals from Invece.Ab, KLOSCAD, SAS, and TIGER 27](#_Toc218859387)

[**Supplementary Table 22.** Study birth year ranges 28](#_Toc218859388)

# **Supplementary methods:** Standardization of outcome scores

First, within each study, raw MMSE scores, pooled across all waves, were transformed

to have a Gaussian (or normal) distribution, calculated so that the transformed value has the same

percentile value as the original value in the original distribution (in SPSS such scores are described

simply as normal scores, but are produced under the Rank Cases procedure). Transformed score

outliers were then winsorized to values plus or minus 3 standard deviations (SDs) from the mean

scores. These were then standardized by converting to *Z*-scores within each study, using estimated

means and SDs of baseline scores within each study at common values of age, sex, and education.

The common values were the average values at baseline from data pooled across all studies (common values: age = 72.7 years, education = 9.0 years, and sex = 0.42, indicating 42% males). SDs used for the calculation of the *Z*-scores were the estimated SDs of the residuals (i.e., the standard errors [SEs] of the estimates) obtained from the regression models for each study after adjustment for age, sex, and education. Our method of standardizing scores from multiple studies is essentially

the same as that described by Griffith L, et al. (Harmonization of cognitive measures

in individual participant data and aggregate data meta-analysis. Methods research report. Rockville

(MD): Agency for Healthcare Research and Quality; 2013) for obtaining standardized demographically based category-centered scores. However, instead of obtaining *Z-*scores using means and SDs from subsamples within each study with the same restricted ranges of demographic characteristics, we used regression models to obtain estimated means and SDs for specific common values of demographic variables.

Note that MoCA scores from TIGER were converted to MMSE scores as per Roalf, D. R., Moberg, P. J., Xie, S. X., Wolk, D. A., Moelter, S. T., & Arnold, S. E. (2013). Comparative accuracies of two common screening instruments for classification of Alzheimer's disease, mild cognitive impairment, and healthy aging. Alzheimers Dement, 9(5), 529-537. <https://doi.org/10.1016/j.jalz.2012.10.001>

# **Supplementary results:** Incident DLB and PDD in the two studies included in the fully adjusted analyses

Across Invece.Ab and KLOSCAD, there were 9 and 4 incident DLB and PDD cases, respectively. DLB/PDD accounted for 5.8% (9/154) and 1.7% (4/238) of all dementia among men and women, respectively, and for 13.7% (5/37), 2.5% (7/272), and 1.2% (1/83) of all dementia among individuals aged 60–69, 70–79 and 80–89 years, respectively. The baseline prevalence of disturbing dreams among incident DLB/PDD cases was 61.5 % (8/13), compared to 27.2% and 24.3% among incident all-cause dementia and AD cases, respectively. The prevalence among individuals who remained dementia free was 23.6%.

# **Supplementary Table 1.** Exclusions and missingness

| **Study** | **Total N^a^** | **Baseline dementia** | **Missing dementia data** | **PD^b^** | **Total excluded** | **In the main analyses** | **Missing covariates^c^** |
| --- | --- | --- | --- | --- | --- | --- | --- |
| BCSA | 1427 | 31 (2.2) | 3 (0.2) | 11 (0.8) | 45 (3.2) | 1382 (96.8) | NA |
| ESPRIT | 1995 | 58 (2.9) | 0 (0.0) | 23 (1.2) | 79 (4.0) | 1916 (96.0) | NA |
| Invece.Ab | 1153 | 33 (2.9) | 9 (0.8) | 30 (2.6) | 65 (5.6) | 1088 (94.4) | 248 (22.8) |
| KLOSCAD^d^ | 5271 | 237 (4.5) | 42 (0.8) | 47 (0.9) | 330 (6.3) | 4941 (93.7) | 799 (16.2) |
| SAS | 644 | 51 (7.9) | 11 (1.7) | 1 (0.2) | 63 (9.8) | 581 (90.2) | NA |
| TIGER | 392 | 56 (14.3) | 0 (0.0) | 6 (1.5) | 62 (15.8) | 330 (84.2) | 19 (5.7) |
| Total | 10882 | 466 (4.3) | 65 (0.6) | 118 (1.1) | 644 (5.9) | 10238 (94.1) | 1066 (16.8)^e^ |

Note: All data are presented as n (%).

^a^ Aged 60-89 years and with disturbing dream data and at least one follow-up assessment.

^b^ Parkinson’s disease (PD) was self-reported in all studies except for BCSA, for which it was considered as positive responses to all nine questions about Parkinson’s symptoms given 4 years after baseline. Twenty-one also had baseline dementia or were missing dementia data.

^c^ Not included in the fully adjusted analyses.

^d^ 12 (0.2%) war veterans were also excluded.

^e^ Denominator across the three relevant studies is 6359.

# **Supplementary Table 2.** Contributing study ethics approvals

| BCSA | Ethics Boards of the Fundac¸a˜o Oswaldo Cruz in Rio de Janeiro and the Instituto Rene´ Rachou of the Fundac¸a˜o Oswaldo Cruz in Belo Horizonte, Brazil (14/2007 - CEPSH-CpqRR) |
| --- | --- |
| ESPRIT | Ethics committee (CCPPRB) of the Kremlin Bicetre hospital (n° registered 99-28) |
| Invece.Ab | Ethics Committee of the University of Pavia (#3/2009) |
| KLOSCAD | Institutional review board of Seoul National University Bundang Hospital (No. B-0912-089-010), |
| SAS | Medical Ethics Committee of _Huashan Hospital, Fudan University, Shanghai, China (approval number: HIRB2009-195) |
| TIGER | Research Ethics Committee of the National Taiwan University Hospital. Approval number: 201101039RB, 201312156RINC, 201412212RINC, 201712220RIN, 202012214RIN. |

# **Supplementary Table 3.** Harmonisation of income

| **Study** | **Income** |
| --- | --- |
| BCSA | 1. <2 min salary (1039 reais) 2. <4 min salary 3. ≥4 min salary |
| ESPRIT | 1. <1524 euro 2. 1524-2286 3. ≥2286 |
| Invece.Ab | 1. “inadequate” 2. “just sufficient” 3. “adequate” |
| KLOSCAD | 1. <800 USD/month 2. 800-2500 3. >2500 |
| SAS | 1. <500 RMB 2. 500-1200 3. >1200 |
| TIGER | 1. <10,000 USD 2. 10000-33333 3. >33333 |

# **Supplementary Table 4.** Harmonisation of depression and anxiety

| **Study** | **History of depression** | **State depression** | **Anxiety** |
| --- | --- | --- | --- |
| BCSA | NA | General Health Questionnaire (GHQ-12) score ≥5 | NA |
| ESPRIT | Lifetime major depressive episode (MINI) | CES-D score ≥16 | Current anxiety disorder, incl. generalised (MINI), or treated with an anxiolytic in the past month |
| Invece.Ab | Clinical diagnosis or antidepressant use | GDS-15 score ≥6 | Hypnotic or anxiolytic use |
| KLOSCAD | History of depressive disorder | GDS-30 score ≥16 (Kim et al. *Psychiatry Investig* 2008;5:232-238) | Anxiety disorder not in "complete remission" |
| SAS | History of depression | CES-D ≥16 | Self-rating Anxiety Scale ≥45 |
| TIGER | Medical treatment or antidepressant use | CES-D ≥16 | Anxiety self-reported as "moderate" or "terrible" (assessed 4 yrs prior) |

CES-D: Center for Epidemiologic Studies Depression Scale

GDS: Geriatric Depression Scale (15- or 30-item versions)

MINI: Mini-International Neuropsychiatric Interview

# **Supplementary Table 5.** Harmonisation of insomnia, snoring, daytime sleepiness, medications for sleep or influencing disturbing dream frequency

| **Study** | **Sleep onset insomnia** | **Sleep maintenance insomnia** | **Snoring** | **Daytime sleepiness** | **Medications** |
| --- | --- | --- | --- | --- | --- |
| BCSA | Difficulty falling asleep, ≥3 times/wk and causing any distress during the previous 30 days | Either early morning awakening or difficulty maintaining sleep ≥3 times/wk and causing any distress during the previous 30 days | In the last month, did you snore or your partner reported that you snored? | Sleepiness ≥3 times/wk in the last month, causing any interference in usual activities | Use of hypnotics |
| ESPRIT | Sleep latency >30 mins | Often wakes in the night or wakes up early in the morning | Snores loudly | Epworth Sleepiness Scale score ≥11 | Antihypertensives, beta blockers, anticholinesterases, antidepressants, hypnotics |
| Invece.Ab | PSQI: cannot get to sleep within 30 mins | PSQI: wake up in the middle of the night or early morning | PSQI: cough or snore loudly | PSQI: trouble staying awake | Antidepressants, hypnotics, anxiolytics, beta-blockers, cholinesterase inhibitors, levodopa |
| KLOSCAD | PSQI: cannot get to sleep within 30 mins | PSQI: wake up in the middle of the night or early morning | PSQI: cough or snore loudly | PSQI: trouble staying awake | Cognitive enhancers, psychotropics, steroids, beta-blockers, choline alfoscerate, hypnotics |
| SAS | PSQI: cannot get to sleep within 30 mins | PSQI: wake up in the middle of the night or early morning | PSQI: cough or snore loudly | PSQI: trouble staying awake | PSQI: use of sleep medicine |
| TIGER | PSQI: cannot get to sleep within 30 mins | PSQI: wake up in the middle of the night or early morning | PSQI: cough or snore loudly | PSQI: trouble staying awake | Antidepressants, antihypertensives, cholinesterase inhibitors, hypnotics |

Note. PSQI questions on insomnia and snoring start with "During the past month, how often have you had trouble sleeping because you ..."; for trouble staying awake the question refers to while driving, eating meals or while engaging in social activity during the past month.

# **Supplementary Table 6.** Harmonisation of cardiovascular disease, hypertension, and diabetes

|  | Cardiovascular disease | Hypertension | Diabetes |
| --- | --- | --- | --- |
| BCSA | Myocardial infarction, angina or atrial fibrillation | BP ≥140/90 mmHg or use of anti-hypertensive medication | Fasting glucose ≥126 mg/dL and/or use of insulin or oral hypoglycaemics |
| ESPRIT | Angina, history of angioplasty, heart operation or myocardial infarction, arrhythmia or atrial fibrillation | BP≥140/90 mmHg or treated or declared | Declared or use of insulin or oral hypoglycaemics |
| Invece.Ab | Myocardial infarction, heart failure, angina, arrhythmia, valvulopathy, coronary heart disease, atrial fibrillation | BP≥130/80 mmHg or use of antihypertensives | FBG ≥126 mg/dL or use of insulin or oral hypoglycemics |
| KLOSCAD | Myocardial infarction, angina, congestive heart failure, arrythmia, or heart surgery | History, self-reported current, or BP≥140/90 mmHg | History, self-reported current, FBG ≥126mg/dL, or non-FBG ≥200mg/dL |
| SAS | Coronary heart disease, myocardial infarction, bypass, valve disease, cardiomyopathy, coronary heart failure, arrythmia, premature beat, atrial fibrillation, pacemaker | Self-reported history | Self-reported history |
| TIGER | Myocardial infarction, coronary heart disease, arrythmia | Self-report or medication | Self-report or medication |

# **Supplementary Table 7.** Harmonisation of physical activity, self-reported health

| **Study** | **Physical activity** | **Self-reported health** |
| --- | --- | --- |
| BCSA | Physical activity = walking for about a kilometre and a half at least 1 to 3 times in the last month, or walked to exercise, done gymnastics or practiced any sport for at least 20 to 30 minutes at least 1 to 3 times a month in the last 90 days; inactivity = no time in the last month, less than once a month or never in the last 3 months. | “Fair”:= poor, “Reasonable”:= fair, “Very good, good”:= good/excellent |
| ESPRIT | Physical activity = walking at least 1 hour a day or sport activity sometimes, regularly or often; inactivity = walking <1 hour a day and sport activity is “never”. | NA |
| Invece.Ab | Physical activity = walking >30 mins, dance, gymnastics, bike, swim, run, tennis, aerobics “1 x week” or more; inactivity = none of these or only walking <30 mins. | “Very bad, pretty bad, so and so”:= poor, “Pretty good”:=fair, “Very good”:=good/excellent |
| KLOSCAD | Activity defined as at least 30 mins per week of light (3MET), moderate (4.5MET). | “Poor”:= poor, “Fair”:= fair, “Good/excellent”:= good/excellent |
| SAS | Physical activity: 3 groups based on MET-min/week. Low (<600), moderate (600 to <1200), high (1200+) as per Laird et al. JAMA Network Open. 2023;6(7):e2322489. | NA |
| TIGER | Physical activity: 3 groups based on MET-min/week. Low (<600), moderate (600 to <1200), high (1200+) as per Laird et al. JAMA Network Open. 2023;6(7):e2322489. | “Very bad, not so good”:= poor, “Average”:= fair, “Good, very good”:= good/excellent |

# **Supplementary Table 8.** Characteristics of individuals either included or not included in the fully adjusted analyses featuring data from three studies

| **Covariate/factor** | **Included (n=5293)** | **Not included (n=1066)** |
| --- | --- | --- |
| Age, mean (SD) | 69.9 (5.7) | 72.3 (6.0)*** |
| Sex (women) | 2911 (55.0) | 659 (61.8)*** |
| Education, mean (SD) | 8.9 (5.1) | 6.1 (4.8)*** |
| Marital Status |  |  |
| Married | 3896 (73.6) | 687 (64.5)*** (n=1065) |
| Widowed | 1166 (22.0) | 334 (31.4) |
| Other | 231 (4.4) | 44 (4.1) |
| Income (ordinal) |  |  |
| 1 | 1494 (28.2) | 440 (42.0)*** (n=1047) |
| 2 | 2409 (45.5) | 437 (41.7) |
| 3 | 1390 (26.3) | 170 (16.2) |
| MMSE z-score, mean (SD) | 0.08 (1.15) | -0.53 (1.27)*** (n=964) |
| Onset insomnia | 3317 (62.7) | 683 (64.2) (n=1064) |
| Maintenance insomnia | 3661 (69.2) | 733 (68.8) |
| Extreme sleep hours | 553 (10.4) | 120 (13.4)** (n=896) |
| Daytime sleepiness | 1788 (33.8) | 445 (41.9)*** (n=1063) |
| Snoring | 1980 (37.4) | 344 (32.3)** |
| Medications | 1140 (21.5) | 231 (21.7) |
| Depression, history | 294 (5.6) | 58 (5.7) (n=1017) |
| Depression, state | 857 (16.2) | 213 (20.8)*** (n=1024) |
| Anxiety | 192 (3.6) | 51 (4.8) (n=1063) |
| Hypertension | 3230 (61.0) | 652 (61.0) (n=1063) |
| Diabetes | 1178 (22.3) | 273 (25.8)* (n=1058) |
| Cardiovascular disease | 711 (13.4) | 146 (13.7) (n=1065) |
| Stroke | 335 (6.3) | 103 (9.8)*** (n=1051) |
| Health |  |  |
| Poor | 848 (16.0) | 275 (26.1)*** (n=1054) |
| Fair | 2599 (49.1) | 500 (47.4) |
| Good/excellent | 1846 (34.9) | 279 (26.5) |
| Physical inactivity | 3070 (58.0) | 768 (72.1)*** (n=1065) |
| BMI (kg/m^2^), mean (SD) | 24.7 (3.5) | 24.9 (4.3) (725) |
| Alcohol | 1815 (34.3) | 311 (29.8)** (n=1045) |
| Smoking | 526 (9.9) | 132 (12.6)* (n=1050) |
| APOE ε4 | 1063 (20.1) | 110 (17.6) (n=625) |

Note. Values are n (%) unless otherwise indicated. Differences between groups were investigated using chi-square or independent sample t tests. Numbers of individuals in the not included group are indicated for variables with missing data.

Abbreviations: APOE ε4, apolipoprotein E ε4 allele; BMI, body mass index; MMSE, Mini-Mental State Examination.

*p<0.05.

**p<0.01.

***p<0.001.

# **Supplementary Table 9.** Availability of disturbing dream, dementia/AD, and complete covariate data across studies

| **Study** | **Disturbing dreams** | | **Incident dementia** | | **Covariates** |
| --- | --- | --- | --- | --- | --- |
|  | Yes/no | <1 or ≥1/wk | All-cause | AD |  |
| BCSA | ✓ |  | ✓ |  |  |
| ESPRIT | ✓ |  | ✓ | ✓ |  |
| Invece.Ab | ✓ | ✓ | ✓ | ✓ | ✓ |
| KLOSCAD | ✓ | ✓ | ✓ | ✓ | ✓ |
| SAS | ✓ | ✓ | ✓ | ✓ |  |
| TIGER | ✓ | ✓ | ✓ |  | ✓ |

# **Supplementary Table 10.** Baseline demographic characteristics of the included participants for each cohort study

| **Study** | **N** | **Age, y, mean (SD)** | **Age range, y** | **Sex, male, %** | **Education, y, mean (SD)** |
| --- | --- | --- | --- | --- | --- |
| BCSA | 1382 | 68.5 (6.7) | 60-89 | 38.3 | 2.8 (3.0) |
| ESPRIT | 1916 | 72.8 (5.2) | 65-89 | 41.0 | 10.1 (3.3) |
| Invece.Ab | 1088 | 72.1 (1.3) | 70-75 | 45.8 | 6.9 (3.3) |
| KLOSCAD | 4941 | 69.5 (6.2) | 60-89 | 43.4 | 8.4 (5.3) |
| SAS | 581 | 70.7 (7.3) | 60-89 | 43.5 | 11.4 (4.1) |
| TIGER | 330 | 75.4 (4.5) | 68-89 | 44.8 | 14.1 (3.3) |

**Supplementary Table 11.** Disturbing dream prevalence across the studies

| **Study** | **Disturbing dream prevalence (n, %)** | |
| --- | --- | --- |
|  | **Any** | **≥1/wk** |
| BCSA | 396 (28.7) | NA |
| ESPRIT | 469 (24.5) | NA |
| Invece.Ab | 295 (27.1) | 54 (5.0) |
| KLOSCAD | 1180 (23.9) | 359 (7.3) |
| SAS | 97 (16.7) | 32 (5.5) |
| TIGER | 40 (12.1) | 16 (4.8) |

# **Supplementary Table 12.** Covariate data for disturbing dream groups (no/yes)

| **Covariate** | **Disturbing dreams** | |
| --- | --- | --- |
|  | **No (n=4040)** | **Yes (n=1253)** |
| Age, mean (SD) | 69.9 (5.7) | 69.8 (5.6) |
| Sex (women) | 2224 (55.0) | 687 (54.8) |
| Education, mean (SD) | 8.9 (5.0) | 9.1 (5.1) |
| Marital Status |  |  |
| Married | 3975 (73.6) | 921 (73.5) |
| Widowed | 883 (21.9) | 283 (22.6) |
| Other | 182 (4.5) | 49 (3.9) |
| Income (ordinal) |  |  |
| 1 | 1149 (28.4) | 345 (27.5) |
| 2 | 1854 (45.9) | 555 (44.3) |
| 3 | 1037 (25.7) | 353 (28.2) |
| MMSE z-score, mean (SD) | 0.07 (1.16) | 0.08 (1.11) |
| Onset insomnia | 2354 (58.3) | 963 (76.9)*** |
| Maintenance insomnia | 2595 (64.2) | 1066 (85.1)*** |
| Extreme sleep hours | 382 (9.5) | 171 (13.6)*** |
| Daytime sleepiness | 1223 (30.3) | 565 (45.1)*** |
| Snoring | 1285 (31.8) | 695 (55.5)*** |
| Medications | 787 (19.5) | 353 (28.2)*** |
| Depression, history | 194 (4.8) | 100 (8.0)*** |
| Depression, state | 515 (12.7) | 342 (27.3)*** |
| Anxiety | 123 (3.0) | 69 (5.5)*** |
| Hypertension | 2474 (61.2) | 756 (60.3) |
| Diabetes | 878 (21.7) | 300 (23.9) |
| Cardiovascular disease | 537 (13.3) | 174 (13.9) |
| Stroke | 255 (6.3) | 80 (6.4) |
| Health |  |  |
| Poor | 572 (14.2) | 276 (22.0)*** |
| Fair | 1954 (48.4) | 645 (51.5) |
| Good/excellent | 1514 (37.5) | 332 (26.5) |
| Physical inactivity | 2326 (57.6) | 744 (59.4) |
| BMI (kg/m^2^), mean (SD) | 24.7 (3.5) | 24.7 (3.5) |
| Alcohol | 1346 (33.3) | 469 (37.4)** |
| Smoking | 394 (9.8) | 132 (10.5) |
| APOE ε4 | 802 (19.9) | 261 (20.8) |

Note. Values are n (%) unless otherwise indicated. Differences between disturbing dream group were investigated using chi-square or independent sample t tests.

Abbreviations: APOE ε4, apolipoprotein E ε4 allele; BMI, body mass index; MMSE, Mini-Mental State Examination.

*p<0.05.

**p<0.01.

***p<0.001.

# **Supplementary Table 13.** Follow-up durations, incidence of dementia and AD, and time from baseline to incident all-cause dementia or AD

| **Age or sex group** | **Maximum follow-up, y** | **Follow-up, y, mean (SD)** | **Dementia incidence^a^** | **Time to dementia, y, mean (SD)** | **AD incidence^a^** | **Time to AD, y, mean (SD)** |
| --- | --- | --- | --- | --- | --- | --- |
| Age, y |  |  |  |  |  |  |
| 60-69 | 17.2 | 7.1 (4.1) | 3.9 | 6.3 (3.9) | 1.2 | 6.0 (4.8) |
| 70-79 | 17.0 | 6.4 (3.7) | 13.9 | 5.0 (3.5) | 7.4 | 4.5 (3.5) |
| 80-89 | 16.5 | 4.7 (3.1) | 41.7 | 3.6 (2.8) | 22.1 | 2.9 (2.1) |
| Sex |  |  |  |  |  |  |
| Female | 16.7 | 6.7 (4.0) | 11.8 | 5.1 (3.6) | 6.1 | 4.5 (3.6) |
| Male | 17.2 | 6.3 (3.7) | 9.5 | 4.4 (3.3) | 4.2 | 3.8 (3.2) |

Abbreviation: AD = Alzheimer dementia.

^a^ Cases per 1000 person-years.

# **Supplementary Table 14.** Follow-up durations and dementia outcomes for the studies

| **Study** | **Max. follow-up, yrs** | **Follow-up, yrs (mean, SD)** | **Incident dementia (n, %)** | **Time to dementia, yrs (mean, SD)** | **Incident AD (n, %)** | **Time to AD, yrs (mean, SD)** |
| --- | --- | --- | --- | --- | --- | --- |
| BCSA | 15.0 | 9.8 (4.7) | 151 (10.9) | 6.6 (2.9) | NA | NA |
| ESPRIT | 17.2 | 9.7 (5.2) | 196 (10.2) | 7.2 (4.4) | 119 (6.5) | 6.9 (4.5) |
| Invece.Ab | 9.1 | 6.5 (2.4) | 108 (9.9) | 4.3 (2.1) | 44 (4.3) | 4.2 (2.1) |
| KLOSCAD | 7.9 | 4.7 (1.6) | 208 (4.2) | 2.6 (1.5) | 162 (3.3) | 2.7 (1.5) |
| SAS | 7.3 | 5.7 (1.1) | 45 (7.7) | 2.4 (0.7) | 32 (5.6) | 2.2 (0.7) |
| TIGER | 5.7 | 3.5 (1.0) | 18 (5.5) | 2.1 (1.0) | NA | NA |

# **Supplementary Table 15.** Risk of incident all-cause and Alzheimer dementia associated with any disturbing dreams: all individuals from BCSA, ESPRIT, Invece.Ab, KLOSCAD, SAS, and TIGER

| **Group** | **Disturbing dreams** | **All-cause dementia^†^** | | | **Alzheimer dementia^‡^** | | |
| --- | --- | --- | --- | --- | --- | --- | --- |
|  |  | **n/n[event]** | **HR (95% CI)** | ***p*-value** | **n/n[event]** | **HR (95% CI)** | ***p*-value** |
| **All** | **No** | 7761/542 | 1.00 (ref) |  | 6339/277 | 1.00 (ref) |  |
|  | **Yes** | 2477/184 | 1.03 (0.87–1.22) | 0.703 | 1987/80 | 0.93 (0.72–1.19) | 0.554 |
| **60–69 y** | **No** | 3381/84 | 1.00 (ref) |  | 2739/25 | 1.00 (ref) |  |
|  | **Yes** | 1064/39 | 1.28 (0.87–1.88) | 0.200 | 800/12 | 1.51 (0.76–3.01) | 0.254 |
| **70–79 y** | **No** | 3740/340 | 1.00 (ref) |  | 3124/185 | 1.00 (ref) |  |
|  | **Yes** | 1238/104 | 0.88 (0.71–1.10) | 0.252 | 1061/51 | 0.81 (0.59–1.11) | 0.192 |
| **80–89 y** | **No** | 640/118 | 1.00 (ref) |  | 476/67 | 1.00 (ref) |  |
|  | **Yes** | 175/41 | 1.35 (0.95–1.93) | 0.097 | 126/17 | 1.03 (0.60–1.76) | 0.917 |
| **Women** | **No** | 4372/349 | 1.00 (ref) |  | 3557/190 | 1.00 (ref) |  |
|  | **Yes** | 1510/116 | 0.94 (0.76–1.16) | 0.567 | 1182/52 | 0.87 (0.64–1.18) | 0.357 |
| **Men** | **No** | 3389/193 | 1.00 (ref) |  | 2782/87 | 1.00 (ref) |  |
|  | **Yes** | 967/68 | 1.24 (0.94–1.64) | 0.117 | 805/28 | 1.10 (0.71–1.69) | 0.649 |

*Note*. CI, confidence interval; HR, hazard ratio; ref, reference group. Models are adjusted for age group and sex.

^†^ Number of studies in the models = 6 (BCSA, ESPRIT, Invece.Ab, KLOSCAD, SAS, TIGER), except for 5 studies (BCSA, ESPRIT, KLOSCAD, SAS, TIGER), with the 60–69 y and 80–89 y group models.

^‡^ Number of studies in the models = 4 (ESPRIT, Invece.Ab, KLOSCAD, SAS), except for 3 studies (ESPRIT, KLOSCAD, SAS), with the 60–69 y and 80–89 y group models.

# **Supplementary Table 16.** Risk of incident all-cause and Alzheimer dementia associated with any disturbing dreams in the past month: all individuals from Invece.Ab, KLOSCAD, and TIGER

| **Group** | **Disturbing dreams** | **All-cause dementia^†^** | | | **Alzheimer dementia^‡^** | | |
| --- | --- | --- | --- | --- | --- | --- | --- |
|  |  | **n/n[event]** | **HR (95% CI)** | ***p*-value** | **n/n[event]** | **HR (95% CI)** | ***p*-value** |
| **All** | **No** | 4844/252 | 1.00 (ref) |  | 4475/158 | 1.00 (ref) |  |
|  | **Yes** | 1515/82 | 0.99 (0.77–1.27) | 0.958 | 1444/48 | 0.91 (0.66–1.26) | 0.583 |
| **60–69 y** | **No** | 2087/14 | 1.00 (ref) |  | 2055/8 | 1.00 (ref) |  |
|  | **Yes** | 622/12 | 2.91 (1.35–6.29) | 0.007* | 613/8 | 3.39 (1.27–9.03) | 0.015* |
| **70–79 y** | **No** | 2434/182 | 1.00 (ref) |  | 2157/109 | 1.00 (ref) |  |
|  | **Yes** | 805/50 | 0.79 (0.58–1.08) | 0.145 | 755/27 | 0.70 (0.47–1.08) | 0.095 |
| **80–89 y** | **No** | 323/56 | 1.00 (ref) |  | 263/41 | 1.00 (ref) |  |
|  | **Yes** | 88/20 | 1.33 (0.80–2.22) | 0.274 | 76/13 | 1.14 (0.61–2.13) | 0.672 |
| **Women** | **No** | 2702/158 | 1.00 (ref) |  | 2501/112 | 1.00 (ref) |  |
|  | **Yes** | 868/45 | 0.84 (0.60–1.17) | 0.310 | 832/29 | 0.78 (0.52–1.18) | 0.244 |
| **Men** | **No** | 2142/94 | 1.00 (ref) |  | 1974/46 | 1.00 (ref) |  |
|  | **Yes** | 647/37 | 1.27 (0.87–1.86) | 0.214 | 612/19 | 1.27 (0.74–2.17) | 0.381 |

*Note*. CI, confidence interval; HR, hazard ratio; ref, reference group. Models are adjusted for age group and sex.

^†^ Number of studies in the models = 3 (Invece.Ab, KLOSCAD, TIGER), except for 2 studies (KLOSCAD, TIGER), with the 60–69 y and 80–89 y group models.

^‡^ Number of studies in the models = 2 (Invece.Ab, KLOSCAD), except for 1 study (KLOSCAD), with the 60–69 y and 80–89 y group models.

**p*<0.05, following adjustment for multiple comparisons within the age and sex groups.

# **Supplementary Table 17.** Risk of incident all-cause and Alzheimer dementia associated with disturbing dreams ≥1/wk in the past month: all individuals from Invece.Ab, KLOSCAD, SAS, and TIGER

| **Group** | **Disturbing dreams** | **All-cause dementia^†^** | | | **Alzheimer dementia^‡^** | | |
| --- | --- | --- | --- | --- | --- | --- | --- |
|  |  | **n/n[event]** | **HR (95% CI)** | ***p*-value** | **n/n[event]** | **HR (95% CI)** | ***p*-value** |
| **All** | **<1/wk** | 6479/341 | 1.00 (ref) |  | 6055/214 | 1.00 (ref) |  |
|  | **≥1/wk** | 461/38 | 1.36 (0.97–1.90) | 0.074 | 432/24 | 1.30 (0.85–1.99) | 0.225 |
| **60–69 y** | **<1/wk** | 2820/25 | 1.00 (ref) |  | 2781/14 | 1.00 (ref) |  |
|  | **≥1/wk** | 171/4 | 2.93 (1.02–8.46) | 0.047 | 167/3 | 3.95 (1.13–13.85) | 0.032 |
| **70–79 y** | **<1/wk** | 3231/234 | 1.00 (ref) |  | 2915/140 | 1.00 (ref) |  |
|  | **≥1/wk** | 241/21 | 1.26 (0.81–1.97) | 0.319 | 225/14 | 1.29 (0.74–2.24) | 0.359 |
| **80–89 y** | **<1/wk** | 428/82 | 1.00 (ref) |  | 359/60 | 1.00 (ref) |  |
|  | **≥1/wk** | 49/13 | 1.37 (0.76–2.46) | 0.295 | 40/7 | 1.03 (0.47–2.25) | 0.932 |
| **Women** | **<1/wk** | 3607/206 | 1.00 (ref) |  | 3377/144 | 1.00 (ref) |  |
|  | **≥1/wk** | 291/24 | 1.27 (0.83–1.94) | 0.273 | 275/15 | 1.11 (0.65–1.89) | 0.699 |
| **Men** | **<1/wk** | 2872/135 | 1.00 (ref) |  | 2678/70 | 1.00 (ref) |  |
|  | **≥1/wk** | 14/170 | 1.57 (0.90–2.73) | 0.109 | 157/9 | 1.91 (0.95–3.85) | 0.072 |

*Note*. CI, confidence interval; HR, hazard ratio; ref, reference group. Models are adjusted for age group and sex.

^†^ Number of studies in the models = 4 (Invece.Ab, KLOSCAD, SAS, TIGER), except for 3 studies (KLOSCAD, SAS, TIGER), with the 60–69 y and 80–89 y group models.

^‡^ Number of studies in the models = 3 (Invece.Ab, KLOSCAD, SAS), except for 2 studies (KLOSCAD, SAS), with the 60–69 y and 80–89 y group models.

# **Supplementary Table 18.** Risk of incident all-cause and Alzheimer dementia associated with disturbing dreams ≥1/wk in the past month: all individuals from Invece.Ab, KLOSCAD, and TIGER

| **Group** | **Disturbing dreams** | **All-cause dementia^†^** | | | **Alzheimer dementia^‡^** | | |
| --- | --- | --- | --- | --- | --- | --- | --- |
|  |  | **n/n[event]** | **HR (95% CI)** | ***p*-value** | **n/n[event]** | **HR (95% CI)** | ***p*-value** |
| **All** | **<1/wk** | 5930/299 | 1.00 (ref) |  | 5516/182 | 1.00 (ref) |  |
|  | **≥1/wk** | 429/35 | 1.43 (1.01–2.03) | 0.045* | 403/24 | 1.50 (0.98–2.30) | 0.063 |
| **60–69 y** | **<1/wk** | 2550/22 | 1.00 (ref) |  | 2513/13 | 1.00 (ref) |  |
|  | **≥1/wk** | 159/4 | 3.18 (1.09–9.27) | 0.034 | 155/3 | 4.06 (1.15–14.34) | 0.030 |
| **70–79 y** | **<1/wk** | 3010/211 | 1.00 (ref) |  | 2699/122 | 1.00 (ref) |  |
|  | **≥1/wk** | 229/21 | 1.37 (0.87–2.15) | 0.170 | 213/14 | 1.44 (0.83–2.51) | 0.199 |
| **80–89 y** | **<1/wk** | 370/66 | 1.00 (ref) |  | 304/47 | 1.00 (ref) |  |
|  | **≥1/wk** | 41/10 | 1.37 (0.70–2.67) | 0.357 | 35/7 | 1.32 (0.60–2.92) | 0.494 |
| **Women** | **<1/wk** | 3300/180 | 1.00 (ref) |  | 3078/126 | 1.00 (ref) |  |
|  | **≥1/wk** | 270/23 | 1.38 (0.95–1.82) | 0.144 | 255/15 | 1.26 (0.74–2.15) | 0.403 |
| **Men** | **<1/wk** | 2630/119 | 1.00 (ref) |  | 2438/56 | 1.00 (ref) |  |
|  | **≥1/wk** | 159/12 | 1.57 (0.97–2.17) | 0.140 | 148/9 | 2.43 (1.20–4.94) | 0.014* |

*Note*. CI, confidence interval; HR, hazard ratio; ref, reference group. Models are adjusted for age group and sex.

^†^ Number of studies in the models = 3 (Invece.Ab, KLOSCAD, TIGER), except for 2 studies (KLOSCAD, TIGER) with the 60–69 y and 80–89 y group models.

^‡^ Number of studies in the models = 2 (Invece.Ab, KLOSCAD), except for 1 study (KLOSCAD) with the 60–69 y and 80–89 y group models.

**p*<0.05, following adjustment for multiple comparisons within the age and sex groups.

# **Supplementary Table 19.** Risk of incident all-cause and Alzheimer dementia associated with disturbing dream frequency in the past month: fully adjusted models with individuals from Invece.Ab, KLOSCAD, and TIGER

| **Group** | **Disturbing dreams** | **Dementia^†^** | |  | **Alzheimer dementia^‡^** | |  |
| --- | --- | --- | --- | --- | --- | --- | --- |
|  |  | **n/n[event]** | **HR (95% CI)** | ***p*-value** | **n/n[event]** | **HR (95% CI)** | ***p*-value** |
| **All** | **<Monthly** | 4040/186 | 1.00 (ref) |  | 3705/111 | 1.00 (ref) |  |
|  | **Monthly** | 916/37 | 0.97 (0.67–1.40) | 0.859 | 880/21 | 0.92 (0.57–1.49) | 0.735 |
|  | **Weekly** | 337/23 | 1.21 (0.76–1.93) | 0.434 | 316/17 | 1.66 (0.95–2.89) | 0.073 |
| **60–69 y** | **<Monthly** | 1849/9 | 1.00 (ref) |  | 1819/4 | 1.00 (ref) |  |
|  | **Monthly** | 426/8 | 4.65 (1.51–14.31) | 0.007 | 421/5 | 6.22 (1.19–32.59) | 0.031 |
|  | **Weekly** | 135/2 | 2.10 (0.35–12.44) | 0.415 | 131/1 | 1.87 (0.13–27.87) | 0.651 |
| **70–79 y** | **<Monthly** | 1978/141 | 1.00 (ref) |  | 1726/83 | 1.00 (ref) |  |
|  | **Monthly** | 457/22 | 0.76 (0.47–1.20) | 0.237 | 429/11 | 0.65 (0.34–1.24) | 0.194 |
|  | **Weekly** | 174/16 | 1.36 (0.79–2.36) | 0.269 | 161/12 | 1.81 (0.95–3.47) | 0.072 |
| **80–89 y** | **<Monthly** | 213/36 | 1.00 (ref) |  | 160/24 | 1.00 (ref) |  |
|  | **Monthly** | 33/7 | 1.15 (0.45–2.93) | 0.763 | 30/5 | 1.15 (0.36–3.71) | 0.814 |
|  | **Weekly** | 28/5 | 0.92 (0.31–2.71) | 0.873 | 24/4 | 1.41 (0.36–5.56) | 0.621 |
| **Women** | **<Monthly** | 2224/115 | 1.00 (ref) |  | 2044/80 | 1.00 (ref) |  |
|  | **Monthly** | 489/18 | 0.77 (0.46–1.29) | 0.314 | 471/13 | 0.77 (0.42–1.43) | 0.413 |
|  | **Weekly** | 198/13 | 1.09 (0.58–2.02) | 0.794 | 188/10 | 1.16 (0.57–2.37) | 0.684 |
| **Men** | **<Monthly** | 1816/71 | 1.00 (ref) |  | 1661/31 | 1.00 (ref) |  |
|  | **Monthly** | 427/19 | 1.27 (0.74–2.19) | 0.385 | 409/8 | 1.46 (0.63–3.39) | 0.377 |
|  | **Weekly** | 139/10 | 1.26 (0.61–2.61) | 0.538 | 128/7 | 4.00 (1.55–10.31) | 0.004* |

*Note.* CI, confidence interval; HR, hazard ratio; ref, reference group. Models are adjusted for age, sex, years of education, marital status, income, sleep onset insomnia, sleep maintenance insomnia, extreme sleep hours, daytime sleepiness, snoring, medications for sleep or that may influence disturbing dream frequency, history of depression, state depression, anxiety, hypertension, diabetes, cardiovascular disease, history of stroke, physical inactivity, current alcohol drinker, current smoker, APOE ε4 allele carriage, MMSE score, body mass index, and self-rated health.

^†^ Number of studies in the models = 3 (Invece.Ab, KLOSCAD, TIGER), except for 2 studies (KLOSCAD, TIGER) with the 60–69 y and 80–89 y group models.

^‡^ Number of studies in the models = 2 (Invece.Ab, KLOSCAD), except for 1 study (KLOSCAD) with the 60–69 y and 80–89 y group models.

**p*<0.05, following adjustment for multiple comparisons within the age and sex groups.

# **Supplementary Table 20.** Risk of incident all-cause and Alzheimer dementia associated with disturbing dream frequency in the past month: all individuals from Invece.Ab, KLOSCAD, and TIGER

| **Group** | **Disturbing dreams** | **Dementia^†^** | |  | **Alzheimer dementia^‡^** | |  |
| --- | --- | --- | --- | --- | --- | --- | --- |
|  |  | **n/n[event]** | **HR (95% CI)** | ***p*-value** | **n/n[event]** | **HR (95% CI)** | ***p*-value** |
| **All** | **<Monthly** | 4844/252 | 1.00 (ref) |  | 4475/158 | 1.00 (ref) |  |
|  | **Monthly** | 1086/47 | 0.82 (0.60–1.12) | 0.211 | 1041/24 | 0.67 (0.44–1.03) | 0.071 |
|  | **Weekly** | 429/35 | 1.39 (0.98–1.98) | 0.071 | 403/24 | 1.41 (0.92–2.17) | 0.115 |
| **60–69 y** | **<Monthly** | 2087/14 | 1.00 (ref) |  | 2055/8 | 1.00 (ref) |  |
|  | **Monthly** | 463/8 | 2.55 (1.07–6.08) | 0.035 | 458/5 | 2.78 (0.91–8.50) | 0.074 |
|  | **Weekly** | 159/4 | 4.07 (1.34–12.41) | 0.014 | 155/3 | 5.37 (1.42–20.35) | 0.013 |
| **70–79 y** | **<Monthly** | 2434/182 | 1.00 (ref) |  | 2157/109 | 1.00 (ref) |  |
|  | **Monthly** | 486/29 | 0.62 (0.42–0.92) | 0.018 | 542/13 | 0.47 (0.26–0.84) | 0.010 |
|  | **Weekly** | 229/21 | 1.26 (0.80–1.98) | 0.310 | 213/14 | 1.29 (0.74–2.25) | 0.376 |
| **80–89 y** | **<Monthly** | 323/56 | 1.00 (ref) |  | 263/41 | 1.00 (ref) |  |
|  | **Monthly** | 47/10 | 1.26 (0.64–2.47) | 0.506 | 41/6 | 0.99 (0.42–2.34) | 0.984 |
|  | **Weekly** | 41/10 | 1.41 (0.90–2.21) | 0.316 | 35/7 | 1.32 (0.59–2.94) | 0.499 |
| **Women** | **<Monthly** | 2702/158 | 1.00 (ref) |  | 2501/112 | 1.00 (ref) |  |
|  | **Monthly** | 598/22 | 0.62 (0.40–0.97) | 0.034 | 577/14 | 0.58 (0.33–1.01) | 0.054 |
|  | **Weekly** | 270/23 | 1.29 (0.58–2.02) | 0.259 | 255/15 | 1.17 (0.68–2.01) | 0.579 |
| **Men** | **<Monthly** | 2142/94 | 1.00 (ref) |  | 1974/46 | 1.00 (ref) |  |
|  | **Monthly** | 488/25 | 1.16 (1.04–2.50) | 0.501 | 464/10 | 0.91 (0.46–1.80) | 0.788 |
|  | **Weekly** | 159/12 | 1.62 (0.88–2.97) | 0.119 | 148/9 | 2.38 (1.16–4.88) | 0.018 |

*Note*. CI, confidence interval; HR, hazard ratio; ref, reference group. Models are adjusted for age group and sex.

^†^ Number of studies in the models = 3 (Invece.Ab, KLOSCAD, TIGER), except for 2 studies (KLOSCAD, TIGER) with the 60–69 y and 80–89 y group models.

^‡^ Number of studies in the models = 2 (Invece.Ab, KLOSCAD), except for 1 study (KLOSCAD) with the 60–69 y and 80–89 y group models.

# **Supplementary Table 21.** Risk of incident all-cause and Alzheimer dementia associated with disturbing dream frequency in the past month: all individuals from Invece.Ab, KLOSCAD, SAS, and TIGER

| **Group** | **Disturbing dreams** | **Dementia^†^** | |  | **Alzheimer dementia^‡^** | |  |
| --- | --- | --- | --- | --- | --- | --- | --- |
|  |  | **n/n[event]** | **HR (95% CI)** | ***p*-value** | **n/n[event]** | **HR (95% CI)** | ***p*-value** |
| **All** | **<Monthly** | 5328/286 | 1.00 (ref) |  | 4952/185 | 1.00 (ref) |  |
|  | **Monthly** | 1151/55 | 0.89 (0.67–1.19) | 0.433 | 1103/29 | 0.73 (0.49–1.08) | 0.121 |
|  | **Weekly** | 461/38 | 1.33 (0.95–1.87) | 0.097 | 432/24 | 1.24 (0.81–1.90) | 0.320 |
| **60–69 y** | **<Monthly** | 2330/15 | 1.00 (ref) |  | 2298/9 | 1.00 (ref) |  |
|  | **Monthly** | 490/10 | 3.15 (1.41–7.01) | 0.005* | 483/5 | 2.63 (0.88–7.85) | 0.083 |
|  | **Weekly** | 171/4 | 4.02 (1.33–12.16) | 0.014 | 167/3 | 5.06 (1.36–18.81) | 0.016 |
| **70–79 y** | **<Monthly** | 2621/200 | 1.00 (ref) |  | 2340/123 | 1.00 (ref) |  |
|  | **Monthly** | 610/34 | 0.68 (0.47–0.98) | 0.043 | 575/17 | 0.56 (0.34–0.93) | 0.025 |
|  | **Weekly** | 241/21 | 1.18 (0.75–1.85) | 0.480 | 225/14 | 1.18 (0.68–2.05) | 0.551 |
| **80–89 y** | **<Monthly** | 377/71 | 1.00 (ref) |  | 314/53 | 1.00 (ref) |  |
|  | **Monthly** | 51/11 | 1.22 (0.65–2.30) | 0.539 | 45/7 | 1.00 (0.45–2.20) | 0.996 |
|  | **Weekly** | 49/13 | 1.40 (0.78–2.53) | 0.263 | 40/7 | 1.03 (0.47–2.27) | 0.933 |
| **Women** | **<Monthly** | 2967/178 | 1.00 (ref) |  | 2760/126 | 1.00 (ref) |  |
|  | **Monthly** | 640/28 | 0.73 (0.49–1.09) | 0.129 | 617/18 | 0.68 (0.47–2.27) | 0.134 |
|  | **Weekly** | 291/24 | 1.21 (0.79–1.85) | 0.384 | 275/15 | 1.05 (0.61–1.79) | 0.853 |
| **Men** | **<Monthly** | 2361/108 | 1.00 (ref) |  | 2192/59 | 1.00 (ref) |  |
|  | **Monthly** | 511/27 | 1.16 (0.76–1.78) | 0.498 | 486/11 | 0.84 (0.44–1.60) | 0.595 |
|  | **Weekly** | 170/14 | 1.62 (0.93–2.84) | 0.093 | 157/9 | 1.85 (0.91–3.76) | 0.088 |

*Note*. CI, confidence interval; HR, hazard ratio; ref, reference group. Models are adjusted for age group and sex.

^†^ Number of studies in the models = 4 (Invece.Ab, KLOSCAD, SAS, TIGER), except for 3 studies (KLOSCAD, SAS, TIGER) with the 60–69 y and 80–89 y group models.

^‡^ Number of studies in the models = 3 (Invece.Ab, KLOSCAD, SAS), except for 2 studies (KLOSCAD, SAS) with the 60–69 y and 80–89 y group models.

**p*<0.05, following adjustment for multiple comparisons within the age and sex groups.

# **Supplementary Table 22.** Study birth year ranges

| **Study** | **Birth year range** |
| --- | --- |
| BCSA | 1906-1937 |
| ESPRIT | 1916-1935 |
| Invece.Ab | 1935-1939 |
| KLOSCAD | 1925-1953 |
| SAS | 1920-1949 |
| TIGER | 1926-1948 |
